# Frailty status as a potential factor in increased postoperative opioid use in older adults

**DOI:** 10.1186/s12877-021-02101-4

**Published:** 2021-03-18

**Authors:** Elizabeth D. Auckley, Nathalie Bentov, Shira Zelber-Sagi, Lily Jeong, May J. Reed, Itay Bentov

**Affiliations:** 1 Ohio State University College of Medicine, Columbus, Ohio, USA; 2 Department of Family Medicine, University of Washington, Harborview Medical Center, Seattle, WA USA; 3 School of Public Health, University of Haifa, 3498838 Haifa, Israel; 4 University of Washington School of Medicine, Seattle, WA USA; 5 Division of Gerontology and Geriatric Medicine, Department of Medicine, University of Washington, WA Seattle, USA; 6 Department of Anesthesiology and Pain Medicine, University of Washington, Seattle, WA USA

## Abstract

**Background:**

Prescription opioids are commonly used for postoperative pain relief in older adults, but have the potential for misuse. Both opioid side effects and uncontrolled pain have detrimental impacts. Frailty syndrome (reduced reserve in response to stressors), pain, and chronic opioid consumption are all complex phenomena that impair function, nutrition, psychologic well-being, and increase mortality, but links among these conditions in the acute postoperative setting have not been described. This study seeks to understand the relationship between frailty and patterns of postoperative opioid consumption in older adults.

**Methods:**

Patients ≥ 65 years undergoing elective surgery with a planned hospital stay of at least one postoperative day were recruited for this cohort study at pre-anesthesia clinic visits. Preoperatively, frailty was assessed by Edmonton Frailty and Clinical Frailty Scales, pain was assessed by Visual Analog and Pain Catastrophizing Scales, and opioid consumption was recorded. On the day of surgery and subsequent hospitalization days, average pain ratings and total opioid consumption were recorded daily. Seven days after hospital discharge, patients were interviewed using uniform questionnaires to measure opioid prescription use and pain rating.

**Results:**

One hundred seventeen patients (age 73.0 (IQR 67.0, 77.0), 64 % male), were evaluated preoperatively and 90 completed one-week post discharge follow-up. Preoperatively, patients with frailty were more likely than patients without frailty to use opioids (46.2 % vs. 20.9 %, *p* = 0.01). Doses of opioids prescribed at hospital discharge and the prescribed morphine milligram equivalents (MME) at discharge did not differ between groups. Seven days after discharge, the cumulative MME used were similar between cohorts. However, patients with frailty used a larger fraction of opioids prescribed to them (96.7 % (31.3, 100.0) vs. 25.0 % (0.0, 83.3), *p* = 0.007) and were more likely (OR 3.7, 95 % CI 1.13–12.13) to use 50 % and greater of opioids prescribed to them. Patients with frailty had higher pain scores before surgery and seven days after discharge compared to patients without frailty.

**Conclusions:**

Patterns of postoperative opioid use after discharge were different between patients with and without frailty. Patients with frailty tended to use almost all the opioids prescribed while patients without frailty tended to use almost none of the opioids prescribed.

## 1. Background

Prescription drug overdose deaths in the United States, where opioids are among the agents implicated, have increased between 1999 and 2010 in parallel with a 4 fold increase in morphine milligram equivalents (MME) per capita of opioid prescribing [1]. The majority of prescription drug overdose deaths occur in patients that are prescribed according to guidelines and many of those are prescribed low dose therapy (less than 100 mg of MME per day) [2]. Consequently, optimizing prescriber practices is critical to ending the opioid epidemic [3]. Improving opioid prescribing in all populations, with emphasis on older adults, is timely as those 65 and older increase as a percentage of the total population. In 2016, 1 in 3 Medicare part D beneficiaries had an opioid prescription, followed by a 17.2 % increase in prescriptions for opioids from 2016 to 2017 [4, 5]. Postoperative pain management in older adults is complex and is further complicated by an increased incidence of side effects due to polypharmacy, age-related changes in pharmacokinetics, and presurgical collateral pain syndromes [6, 7]. While current guidelines for postoperative pain therapy suggest non-opioid and non-pharmacologic therapies as first line (when tolerable and appropriate), with short term, low dose opioid prescriptions as second line [8], opioids are still commonly provided to older adults during the postoperative period [9] despite low efficacy in many patients [10]. Inappropriate prescribing of opioids, defined as “inadequate, continued, or excessive prescribing that poses high risks of morbidity and mortality”[11], may lead to a marked increase in the prevalence of opioid misuse [12]. Opioid misuse may impact not only the patients for whom the opioids were prescribed, but also family members and caretakers, since opioids and other pharmacotherapies left unsecured in the home are a potential source for inappropriate use [13]. Ideally, a postoperative prescription of opioids and other pharmacotherapies after discharge from the hospital would be sufficient to effectively alleviate pain, but leave minimal amounts of unused opioids that may be subsequently misused.

Aging is accompanied by cumulative declines across multiple physiologic systems, which may result in frailty. Frailty is defined by decreased reserve and resistance to stressors and increased vulnerability to adverse outcomes [14]. Frailty is accompanied by a multi-dimensional decline in domains such as physical performance, reduced gait speed and mobility, impaired nutritional status, and cognitive disorders [15]. Patients with frailty have a higher likelihood of mortality, morbidity, and complications after surgical procedures [16] and may more frequently use chronic analgesics and opioids to mitigate these outcomes [10]. While pain, chronic opioid consumption, and frailty have much in common as multidimensional conditions that impact function, nutrition, psychologic well-being, and ultimately mortality, relatively little research linking acute opioid consumption with these conditions has been published [17]. The goal of this study is to compare patterns of post-surgical opioid utilization between frail and non-frail older adults.

## 2. Methods

### 2.1 Study design and Population

A prospective cohort study of older surgical patients was conducted over six months during 2018 and 2019 in a single academic institution that serves as both a Level 1 Trauma Center and a safety net hospital (Harborview Medical Center, Seattle, Washington). This study was approved by the institutional review board at the University of Washington (IRB# STUDY00002781) and follows the STROBE guidelines. Patients 65 years and older undergoing elective surgery and anticipated to be admitted to the hospital for an overnight stay were recruited during their visit to the pre-anesthesia clinic. After providing written informed consent, patients were evaluated in the pre-anesthesia clinic before surgery, followed daily during their hospitalization after surgery, and interviewed seven days after their discharge from the hospital. Demographic data was extracted from the electronic medical record.

### 2.2 Assessment of opioid consumption

**Fig. 1:**
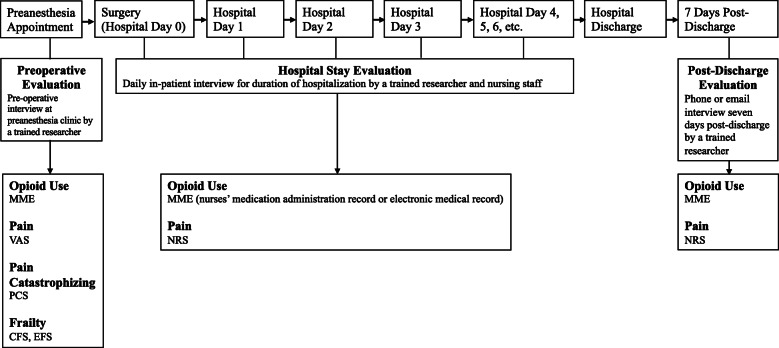
Time course of the study. Patients were interviewed for a preoperative evaluation during their pre-anesthesia clinic appointment. Preoperative evaluation included Visual Analog Scale pain rating, Clinical Frailty Scale and Edmonton Frailty Scale assessments, recorded MME of opioid use, and Pain Catastrophizing Scale assessment for pain catastrophizing. All patients were hospitalized after surgery for at least one postoperative day. On each day of the postoperative hospital stay, pain ratings were obtained from nurses' medical administration records or electronic medical records as a Numerical Rating Score or Verbal Rating Scale, and opioid use was captured in MME from the electronic medical record. Seven days after discharge from the hospital, patients reported pain (using the Numerical Rating Score) and opioid use (in MME) during a phone or email interview. Abbreviations: CFS, Clinical Frailty Scale; EFS, Edmonton Frailty Scale; VAS, Visual Analog Scale; PCS, Pain Catastrophizing Scale; MME, morphine milligram equivalents; NRS, Numerical Rating Score; VRS, Verbal Rating Scale

Preoperative opioid use was evaluated by patients' self-report. Electronic medical records and nurses’ medication administration records were used to track daily opioid administration for each day of hospital stay and opioid prescriptions at discharge (Fig. 1). Opioid prescribing is at the discretion of the physicians, but templates for older adults are available in some portions of the hospital through the electronic medical record [18]. Other medications for pain, such as gabapentin, ketamine, marijuana, NSAIDs, and over the counter medications were also recorded at each timepoint. One week after discharge from the hospital, patients were interviewed by phone or by email to measure total opioid consumption using the prompt “for the (medication) you were prescribed, of (number) of pills of (strength), at discharge, as of seven days after discharge, how many of those pills have you taken?”. All opioid usage was converted into MME (Table 1) [19]. Since reduction of opioid over-prescription is pivotal, the percentage of opioid consumption after discharge from hospitalization was calculated by dividing the amount of MME used by the patient one week after hospital discharge by the total amount of MME prescribed at hospital discharge [20]. Our primary outcome was over-prescription of opioids. Over-prescription was defined by use of less than 50 % of the opioids prescribed at discharge one week after discharge.

**Table 1:** MME Conversion and Opioid Usage Frequency. Factors commonly used to convert opioid doses into morphine milligram equivalents (MME) [18] were used in the study. Preoperative prescription frequency describes the percent of total preoperative opioid prescriptions. Post discharge prescription frequency describes the percent of total discharge prescriptions for each specific drug in this study

Prescription Opioid	Conversion Factor to MME [18]	Preoperative Prescription Frequency (%) (n = 41)	Post discharge Prescription Frequency (%) (n = 87)
Codeine	0.15	2.4	0
Hydrocodone^a^	1	12.2	2.1
Hydromorphone	4	7.3	16.1
Methadone	4	4.9	1.1
Morphine	1	9.8	1.1
Oxycodone^b^	1.5	41.5	79.3
Oxymorphone	3	0	0
Tramadol	0.1	17.1	1.1

^a^Hydrocodone includes all hydrocodone and Vicodin prescriptions

^b^Oxycodone includes all oxycodone and Percocet prescriptions

### 2.3 Assessment of pain

Pain catastrophizing was assessed using the Pain Catastrophizing Scale (PCS) during preoperative evaluation by a trained researcher (Fig. 1). The PCS evaluates pain coping behavior. It consists of 13 items in three categories (magnification, rumination, and helplessness) and is reported as a score between 0 and 52 [21–24]. Pain was scored using an 11-point scale (0–10, where 0 is the absence of pain and 10 is the most severe pain) [25–27]. Preoperative pain was assessed during the pre-anesthesia patient interview by a trained researcher using the visual analog scale (VAS) [28]. During hospitalization, the nurses' medical administration records utilized numerical rating score (NRS) to assess pain (Fig. 1). If several pain scores were collected in one day, the average pain score was calculated. Seven days after hospital discharge, pain was assessed over the phone or email using the numerical rating score. Both numerical rating score and verbal rating score are comparable to the visual analog scale and are validated as translatable to a numerical scale [26, 27].

### 2.4 Preoperative evaluation of Frailty

Frailty was assessed preoperatively by a trained researcher utilizing the Clinical Frailty Scale (CFS) (Fig. 1). The CFS scores frailty as one of nine categories, ranging from 1 (very fit) to 9 (terminally ill), and scores of 5 and above are considered frail. The CFS is accompanied by description and pictographs for each category and requires less than 1 minute to perform [29–31]. The CFS was used since it is simple, highly feasible, and strongly correlated to postoperative outcomes [18, 32]. To validate the use of a simple frailty tool in our cohort, we also evaluated frailty preoperatively using a comprehensive tool, the Edmonton Frailty Scale (EFS), administered by a trained researcher (Fig. 1). The EFS consists of nine domains, including tasks to perform and questions for the patient to answer, and results in a score between 0 and 17, where 8 or higher indicates frailty [33]. The EFS requires patient participation and about 10–15 minutes to administer [34].

### 2.5 Statistical analysis

Statistical analyses were performed using SPSS version 25 (IBM-SPSS Armonk, NY). We tested all variables for normality and found them to be not normally distributed. Continuous variables are presented as median and interquartile range (IQR). To test the differences in continuous variables between two groups, with frailty and without frailty, the Mann–Whitney U test was performed. To compare differences between two groups, with frailty and without frailty, for categorical variables (e.g. with frailty/without frailty and using 50 % and greater of the opioids prescribed) we performed the Pearson’s Chi-Square test. The Spearman correlation was performed to test correlation between different frailty scores. A multivariate logistic regression analysis was performed to compare between the groups with frailty and without frailty in using 50 % and greater of the opioids prescribed with adjustment for age and sex. Odds ratio (OR) and 95 % confidence interval (CI) are presented. *P* value of < 0.05 was considered statistically significant for all analyses.

## 3. Results

**Fig. 2:**
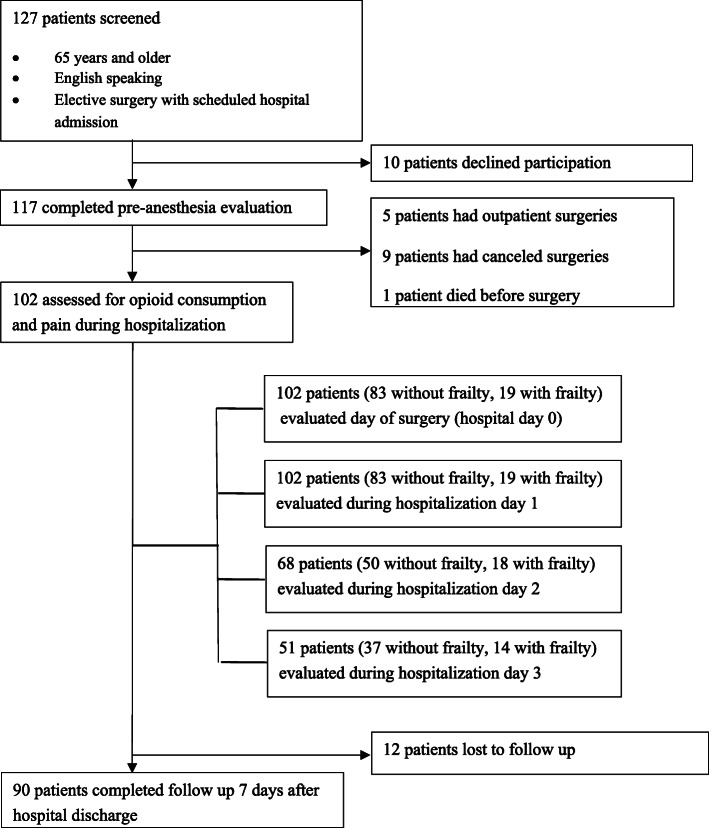
Recruitment and follow-up of patients screened during their visit to the pre-anesthesia clinic. One hundred and twenty-seven patients that presented at the pre-anesthesia clinic during the study period were eligible and screened for participation. A pre-anesthesia evaluation was completed for 117 patients, and 102 patients were followed through their surgery and hospital stay. Follow-up seven days after hospital discharge was completed in 90 patients

One hundred twenty-seven patients were approached in the pre-anesthesia clinic; 117 patients were recruited for the preoperative evaluation. Ninety participants completed the one-week post discharge follow-up with hospital stays ranging from 1 to 26 days (Fig. 2). Of those who completed the preoperative evaluation, 26 patients had frailty and 91 were without frailty as defined by a CFS score of ≥5. Sixteen patients had frailty and 101 were without frailty when using an EFS score of ≥8. The CFS and EFS are well correlated within our sample (r = 0.665, *p*≤0.001). The median age of the population with and without frailty was not different (76.0 vs. 72.0, *p *= 0.20), but the population with frailty was more likely to be female (54 % vs. 31 %, *p* < 0.03). Preoperatively, a greater percentage of patients with frailty used opioids (46.2 % vs. 20.9 %, *p* = 0.01). Of opioids used preoperatively, oxycodone (41.5 %), tramadol (17.1 %), hydrocodone (12.2 %) were used most commonly, and codeine, fentanyl, hydromorphone, methadone, and morphine each made up < 10 % of prescriptions (Table 1). Patients with frailty reported higher pain scores (7.0 (2.5, 8.0) vs. 3.0 (0.5, 7.0), *p* = 0.01) as well as pain catastrophizing scores (13 (5.75, 21.5) vs. 4 (1.0, 11.0), *p* = 0.001). Surgical procedures were classified as spine (*n* = 42), vascular (*n* = 18), neurosurgery (*n* = 20), orthopedic (*n* = 18) (foot and ankle (*n* = 13), femur (*n* = 4), and humerus (*n* = 1)), and other (all those with less than 10 % occurrence: otolaryngology-head and neck, general surgery, maxillofacial, burn plastics, urology, and general surgery) (*n* = 19) (Table 2).

**Table 2:** Description of the Study Participants (median (IQR), unless otherwise stated)

	**All Participants (*n*=117)**	**CFS With Frailty (*n*=26)**	**CFS Without Frailty (*n*=91)**	***P * Value**
**Age (years)**	73.0 (67.0, 77.0)	76.0 (67.0, 81.0)	72.0 (67.0, 77.0)	0.20
**Gender (men %)**	Male: 64%	Male: 46%	Male: 69%	0.03
**Preoperative opioid use (% (n))**	26.5% (31)	46.2% (12)	20.9% (19)	0.01
**PCS**	6.0 (1.0, 13.0)	13.0 (5.8, 21.5)	4.0 (1.0, 11.0)	0.01
**VAS**	4.0 (1.0, 4.0)	7.0 (2.5, 8.0)	3.0 (0.5, 7.0)	0.01
**Length of Stay (days)**	3.0 (1.0, 6.0)	5.0 (2.0, 6.0)	2.0 (1.0, 5.0)	<0.01
**Surgery Type (%)**				
Surgical Spine	36%	42%	34%	
Vascular	15%	12%	16%	
Neurosurgery	17%	15%	18%	
Orthopedic	15%	19%	14%	
Foot and Ankle	11%	4%	13%	
Femur and Humerus	4%	15%	1%	
Other^a^	16%	12%	18%	

Description of the study participants in cohorts with and without frailty. Information was recorded during preoperative patient interviews using validated and informal questionnaires and is presented as median and IQR. The cohorts were defined using a CFS score of <5 as without frailty, and greater than or equal to 5 as with frailty. The cohort with frailty had fewer males, more common preoperative opioid use, higher pain catastrophizing, and more pain preoperatively. Abbreviations: *SD *Standard deviation, *CFS *Clinical Frail Scale, *PCS *Pain Catastrophizing, *VAS* Visual Analog Scale. ^a^Other includes all procedures that occurred in less than 10% of patients: otolaryngology-head and neck, general surgery, oral and maxillofacial, burn plastic, and urology.

### 3.1 Opioid dose requirements and Frailty

**Fig. 3:**
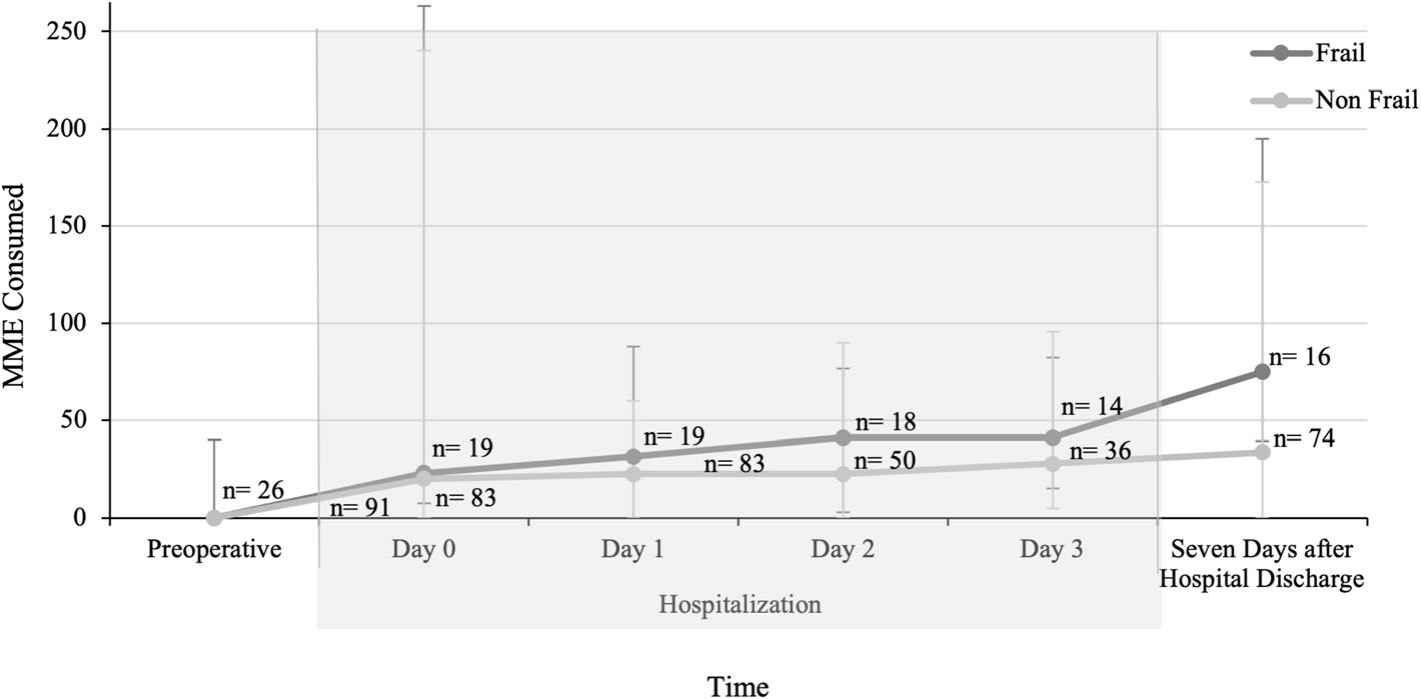
MME median (IQR) usage for patients with and without frailty. Preoperative assessment of MME was performed at the pre-anesthesia clinic. Hospitalization Day 0 is the day of surgery. Hospitalization ranged from 1 to 26 days and assessment was collected from the electronic medical record. The number of patients is reduced in consecutive days because patients were discharged from the hospital. The number of patients who stayed more than 3 days after surgery was small and therefore not presented. Assessment after hospital discharge was conducted by patient self-report. There was no significant difference in the median amount of opioids used during hospitalization or after hospital discharge between patients with and without frailty

There was great variability in quantity of opioids used in both patients with and without frailty. Although median preoperative opioid consumption was zero in both groups, since less than half of both study populations consumed any opioids preoperatively (Table 2), patients with frailty used larger quantities of opioids preoperatively as measured by MME than patients without frailty (0.0 (0.0, 40.13) vs. 0.0 (0.0, 0.0), *p* = 0.007) (Fig. 3). During hospitalization, the amounts of opioids used were not different between patients with and without frailty: on the day of surgery (23.0 (7.5, 263.0) vs. 30.0 (0.0, 240.0), *p* = 0.493), hospital day 1 (31.5 (0.0, 88.0) vs. 22.5 (0.0, 60.0), *p* = 0.507), hospital day 2 (41.3 (2.8, 76.8) vs. 22.5 (0.0, 90.0), *p* = 0.644), and hospital day 3 (41.3 (15.0, 82.5) vs. 27.8 (4.8, 95.6), *p* = 0.494). In the hospital, oxycodone (33.6 %), fentanyl (23.2 %), hydromorphone (18.1 %), and morphine (15.1 %) were ordered most commonly, with methadone and tramadol each being ordered < 10 % of the time. All patients with frailty (100 %) and most patients without frailty (82.9 %) were discharged from the hospital with an opioid prescription. Discharge prescriptions were most commonly oxycodone (79.3 %) and hydromorphone (16.1 %), with hydrocodone, methadone, morphine, and tramadol each prescribed < 10 % of the time (Table 1). The prescribed MME at discharge was not significantly different between populations (150.0 (90.0, 225.0) vs. 176.25 (52.5, 303.75), *p* = 0.906). Seven days after discharge, the total consumed MME did not significantly differ in patients with and without frailty (75.0 (39.4, 195.0) vs. 33.8 (0.0, 172.5), *p* = 0.129). However, patients with frailty used a larger fraction of the total opioids prescribed to them (96.7 % (31.3, 100.0) vs. 25.0 % (0.0, 83.3), *p* = 0.007).

### 3.2 Patterns of opioid use after Discharge and Frailty

**Fig. 4:**
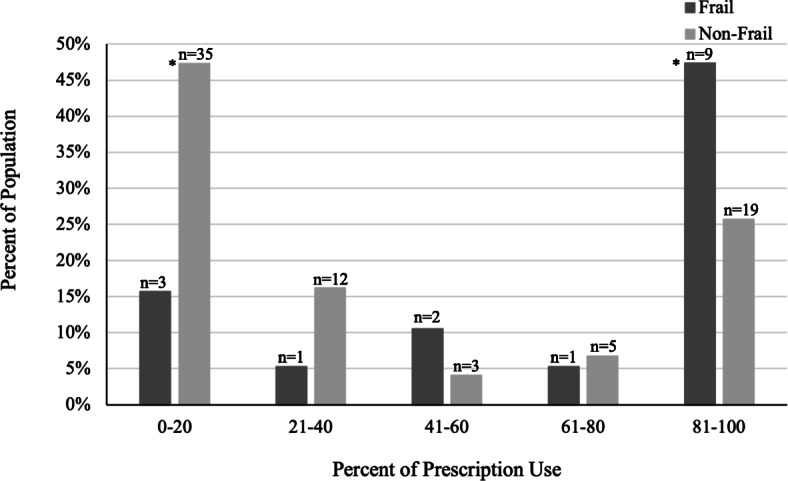
Percent of MME of opioid prescription consumed seven days after hospital discharge. Patients were interviewed by phone or email seven days after hospital discharge to determine how much of their opioid prescription was used. Opioid usage and prescription were recorded in MME and the usage was converted to a percent of the total prescribed opioids. Patients without frailty were more likely to use less than 20 % of the opioid prescription (47.3 % vs. 18.8 %, *p* = 0.036). Patients with frailty were more likely to use 50 % and greater of the opioid prescription (68.8 % with frailty vs. 36.5 % without frailty, *p* = 0.018). *p < 0.05

The primary outcome of our study was the use of less than 50 % of opioid prescriptions by patients seven days after hospital discharge (Fig. 4). Ninety patients (16 with frailty and 74 without frailty) had completed follow-up one week after hospital discharge. It was common for patients in both groups to use almost all or none of their prescriptions. Our results suggest that the pattern of usage was significantly different between patients with and without frailty (Fig. 4). The patients with frailty used 50 % and greater of their prescription more often than the patients without frailty (68.8 % with frailty vs. 36.5 % without frailty, *p* = 0.018). After adjusting for sex and age, the comparison of frailty with 50 % and higher consumption remained significant (OR 3.70, 95 % CI 1.13–12.13). Furthermore, the frail population used greater than 80 % of their prescription more often than the non-frail (56.4 % frail vs. 25.7 % non-frail, *p* = 0.017). The small sample size did not allow for further adjustments (as indicated by the wide CI). In contrast, the patients without frailty were more likely to use less than 20 % of their prescription (47.3 % non-frail vs. 18.8 % frail, *p* = 0.036), an average over prescription of 164.8 MME which is the equivalent of twenty-one 5 mg oxycodone pills.

### 3.3 Pain and frailty

**Fig. 5:**
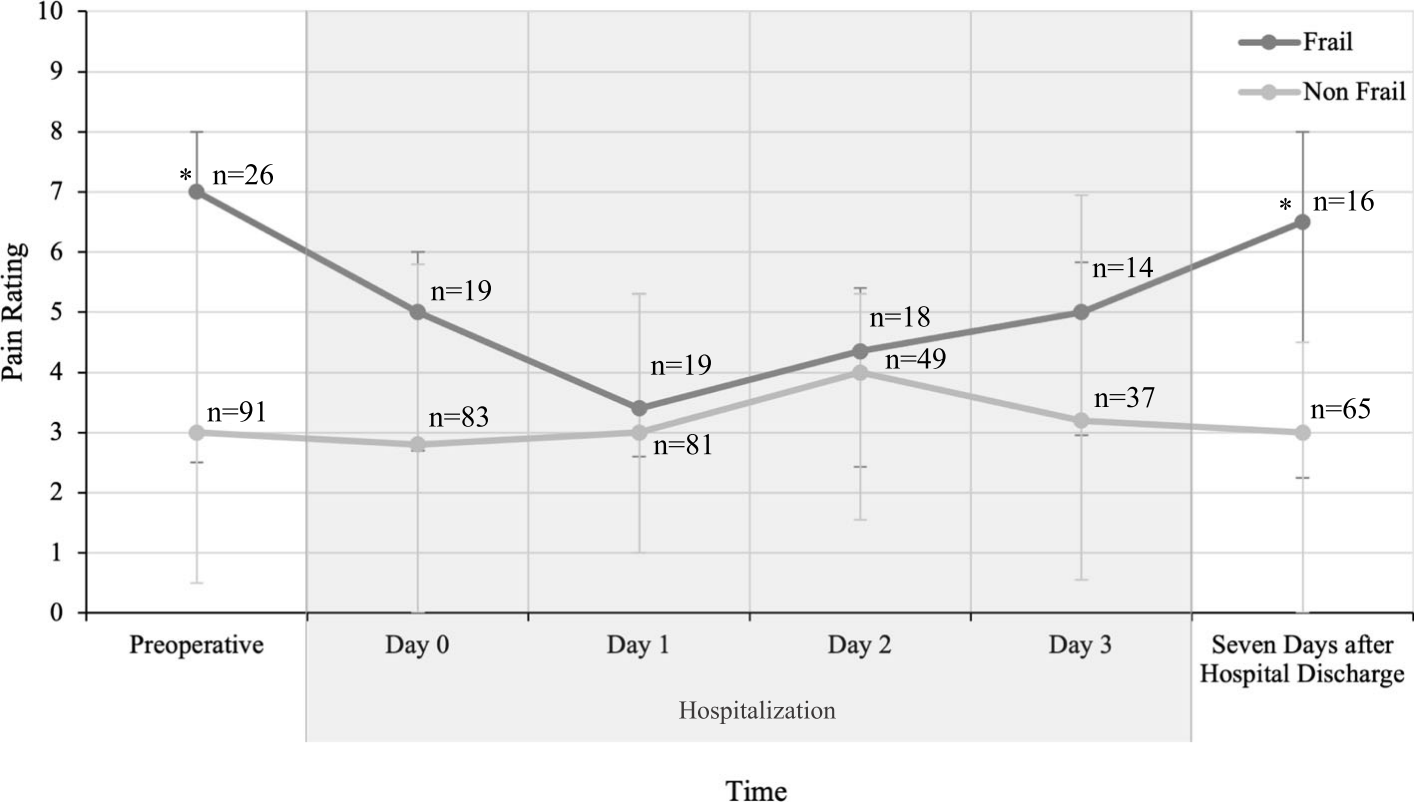
Pain ratings for frail and non-frail patients using comparable 11-point pain scales. Preoperative assessment was done at the pre-anesthesia clinic using the visual analog scale. Hospitalization ranged from 1 to 26 days and assessment was done using numerical rating score and verbal rating score. Assessment after hospital discharge used numerical rating score. Hospitalization Day 0 pain scores are after surgery on the day of surgery. Patients with frailty had more pain 7 days after hospital discharge (6.5 vs. 3.0, *p* < 0.05) compared to patients without frailty, but patients had no differences in pain scores on the day of surgery or during hospitalization. *p < 0.05

Uncontrolled pain after surgery can lead to readmission and may be a risk factor in developing chronic pain [35, 36], while successful pain management interventions often increase appetite, mobility, and function [37]. Preoperatively, patients with frailty reported higher pain scores (7.0 (2.5, 8.0) vs. 3.0 (0.5, 7.0), *p* = 0.01), however; during their hospitalization pain scores were similar. Seven days after discharge from the hospital, pain scores were different again: patients with frailty reported higher pain scores than patients without frailty (6.5 (1.5, 4.25) vs. 3 (1.5, 3), *p* = 0.01) (Fig. 5).

## 4. Discussion

Assessing frailty before surgery is recommended for all older adults [38], and can lead to specific perioperative interventions [39]. Frailty assessment predicts the risk of mortality and reduced independence after surgery, which can inform goals of care discussions [40]. Due to the high sensitivity and the proven utility and feasibility of the CFS in the perioperative setting, we categorized frailty according to the CFS. We found a strong correlation between assessment of frailty using CFS and the more comprehensive EFS. Our results suggest that the simple, quick CFS assessment before surgery can inform patterns of opioid use after discharge from the hospital; patients with frailty use almost all the opioids prescribed to them at hospital discharge while patients without frailty tend to be overprescribed opioids.

Our findings are in agreement with others regarding pain in community dwelling older adults — patients with frailty suffer from more pain (and more intrusive pain) than patients without frailty [41, 42]. Moreover, chronic, persistent, somatic, and osteoarthritic pain are associated with frailty [43, 44]. In our study, patients with frailty reported more pain and higher pain catastrophizing scores preoperatively, but did not report higher pain scores during their hospitalization. When examining the relationship between pain intensity and opioid consumption, pain catastrophizing has been described as a strengthening, but not causational, factor [45].

Opioid doses were variable, and patients with and without frailty did not differ in the amount of opioid medications used in the hospital, or the amount provided at discharge. The novel finding in this study is that frailty is also associated with a unique pattern of opioid use after hospital discharge. First, opioid use in our cohort does not follow a bell-shaped normal distribution. In fact, most patients use very little or almost all of the opioids prescribed. Second, the pattern of discharge opioid use was very different between patients with frailty (about half using almost all the opioid medications that were prescribed to them on discharge from the hospital and less than 20 % used none), and patients without frailty (about half used almost none of the medication prescribed to them and 25 % used almost all). The findings of this study could potentially be explained by greater familiarity with opioid use in the population with frailty (who are more commonly prescribed opioids before surgery), lower tolerance for postoperative pain or reduced coping in patients with frailty (as suggested by higher pain catastrophizing scores before surgery), or more caregiver encouragement for use of pain medication in patients with frailty. Greater opioid usage in patients with frailty may put them at increased risk for side effects of their prescription and subsequent functional difficulties [46]. Conversely, patients without frailty use less of their prescription, which leaves more opioids at home and at possible risk for misuse or abuse by others unless locked in a secure location. Patient characteristics are important in creating evidence-based opioid-prescribing recommendations [47]; it is possible that frailty assessment is an overlooked factor that can be implemented to improve and optimize opioid prescribing.

Patient use of opioids is often reflective of the amount prescribed [20]. Opioid prescriptions should aim to match the minimum amount of anticipated need rather than routine prescribing patterns. Perhaps post discharge opioid prescribing should be based on more frequent assessments, in a manner similar to the shorter intervals now used for pain monitoring during postoperative hospital stays. Additionally, whereas acetaminophen is widely used, the judicious addition of preoperative and postoperative NSAIDs [10, 48] with opioids could potentially further reduce the amount of opioids prescribed.

This study has several limitations: the study population is a small cohort of elective surgical patients at a single institution that is an academic, urban, safety net hospital. Furthermore, opioid use postoperatively in the United States may be higher than in other countries [49], which limits the generalizability of the results. In addition, patients with and without frailty might have had different types of surgeries, or intraoperative management that could result in different requirements for postoperative opioids. The fact that pain scores during the hospitalization and at discharge did not differ between both groups makes this less of a concern. We also note that a sample size calculation was not performed. It is possible that poly-modal medications provided in the perioperative period may interact with other medications that are metabolized by cytochrome p450 and may impact analgesic drug effects. This study does not account for these potential interactions. In addition, there was a significant sex difference between the patients with and without frailty (the percentage of females was significantly higher in the frail group). This sex-related difference in the prevalence of frailty is expected [50]. Lastly, we used patient reported use of opioids after discharge, leading to potential reporting bias. A pill count was encouraged, but not verified, as follow up was limited to a phone interview.

## 5. Conclusions

A simple, rapid frailty assessment may be useful in predicting patterns of opioid use after elective surgery in older adults.

## Data Availability

The datasets generated and analyzed for this study are not publicly available due to privacy concerns. As a small study at a single institution, participant identity may be discernable from demographic information in the data set. The datasets are available from the corresponding author on reasonable request.

## Abbreviations

CFS: Clinical Frailty Scale
EFS: Edmonton Frailty Scale
MME: Morphine milligram equivalents
NRS: Numerical Rating Score
PCS: Pain Catastrophizing Scale
VAS: Visual Analog Scale
VRS: Verbal Rating Scale
